# The prevalence of brucellosis and bovine tuberculosis in ruminants in Sidi Kacem Province, Morocco

**DOI:** 10.1371/journal.pone.0203360

**Published:** 2018-09-18

**Authors:** Hind Yahyaoui Azami, Marie J. Ducrotoy, Mohammed Bouslikhane, Jan Hattendorf, Mike Thrusfield, Raquel Conde- Álvarez, Ignacio Moriyón, Amaia Zúñiga-Ripa, Pilar M. Muñoz Álvaro, Virginie Mick, Ward Bryssinckx, Sue C. Welburn, Jakob Zinsstag

**Affiliations:** 1 Department of Veterinary Pathology and Public Health, Agronomic and Veterinary Institute Hassan II, Rabat, Maroc; 2 Department of Epidemiology and Public Health, Swiss Tropical and Public Health Institute, Basel, Switzerland; 3 University of Basel, Basel, Switzerland; 4 Division of Infection and Pathway Medicine, Centre for Infectious Diseases, School of Biomedical Sciences, College of Medicine and Veterinary Medicine, The University of Edinburgh, Edinburgh, United Kingdom; 5 Royal (Dick) School of Veterinary Studies, The University of Edinburgh, Easter Bush Veterinary Centre, Roslin, Midlothian, United Kingdom; 6 IDISNA - Instituto de Salud Tropical y Depto. Microbiología y Parasitología, Universidad de Navarra, Edificio de Investigación, Pamplona, Spain; 7 Unidad de Producción y Sanidad Animal, Instituto Agroalimentario de Aragón– IA2 - (CITA-Universidad de Zaragoza), Zaragoza, Spain; 8 Paris-Est University/Anses, EU/OIE/FAO & National Reference Laboratory for brucellosis, Animal Health Laboratory, Maisons-Alfort, France; 9 Avia-GIS, Zoersel, Belgium; East Carolina University Brody School of Medicine, UNITED STATES

## Abstract

Bovine tuberculosis (BTB) and brucellosis are major endemic zoonoses in ruminants in Morocco that impact on both animal and human health. This study presents an assessment of the epidemiological and socioeconomic burden of bacterial zoonoses in Sidi Kacem Province in Northern Morocco from a cross-sectional survey of 125 cattle and/or small ruminant-owning households. In total, 1082 sheep and goats were examined from 81 households. The single intradermal comparative cervical test to screen for bovine tuberculosis was undertaken on 1194 cattle from 123 households and all cattle were blood sampled. Cattle and small ruminant sera were tested for brucellosis using the standard Rose Bengal Test (sRBT) and the modified Rose Bengal Test (mRBT). Bacteriology was performed on 21 milk samples obtained from cattle that were seropositive for brucellosis for isolation and phenotyping of circulating *Brucella* strains. Individual and herd prevalence for BTB in cattle of 20.4% (95% CI 18%-23%) and 57.7% (95% CI 48%-66%), respectively, were observed in this study. The prevalence of brucellosis in cattle at individual and herd level was 1.9% (95% CI 1.2%-2.8%) and 9% (95% CI 4.5%-1.5%), respectively. *Brucella* pathogens were isolated from three cattle milk samples and were identified as *B*. *abortus* using Bruceladder^®^ multiplex PCR and *B*. *abortus* biovar 1 by classical phenotyping. All small ruminants were seronegative to sRBT, two were positive to mRBT. A higher risk of BTB and brucellosis was observed in cattle in intensive livestock systems, in imported and crossed breeds and in animals from larger herds (>15). The three risk factors were usually present in the same herds, leading to higher transmission risk and persistence of both zoonoses. These results highlight the importance of implementing control strategies for both BTB and brucellosis to reduce productivity losses and the risk of transmission to humans. Prioritising control for BTB and brucellosis in intensive livestock production systems is essential for human and animal health.

## Introduction

Bovine tuberculosis (BTB) and brucellosis are bacterial zoonoses endemic in cattle, and brucellosis is endemic in small ruminants, in Morocco. These diseases are prioritized in Moroccan veterinary legislation [1,2] but remain poorly controlled. Infectious diseases impose a heavy financial burden on the livestock sector [3] and zoonoses have a dual impact through human disease burden and productivity losses of livestock, on which rural families depend for their livelihoods [4]. While brucellosis and BTB have been controlled and/or eliminated in many developed countries [5,6], in developing nations, these diseases are neglected [7] with the World Health Organization considering control of zoonotic tuberculosis to be a major priority [8]. Rapid growth and intensification of livestock systems is expected to result in an increase in prevalence of both brucellosis and BTB [9].

The infectious agent of bovine BTB is *Mycobacterium bovis*, a member of the *Mycobacterium tuberculosis* complex. Despite a host preference for cattle [10], *M*. *bovis* can infect a wide range of domestic and wild animals [11,12]. Cattle to cattle transmission occurs via direct contact (aerosols) and depends on a number of factors including the number of bacilli excreted and herd density [13]. Transmission of BTB to humans occurs mainly through consumption of infected raw milk, although direct transmission can occur [14]. Bovine tuberculosis is still common in Morocco. A national tuberculosis survey in cattle in 2004, using the single intradermal tuberculin test, showed individual and herd prevalence of 18% (n = 13021) and 33% (n = 2263), respectively [15]. Bovine tuberculosis is responsible for meat losses due to carcass condemnation, and causes a decrease in herd productivity and milk yields [16]. In Morocco, Government BTB control initiatives include tuberculin testing and the slaughtering of positive animals (reactors). At national level, the mandatory BTB control strategy is for test and slaughter but legislation is poorly enforced and no programme of systematic BTB screening of cattle is in place [2,17].

Brucellosis is caused by gram-negative bacteria of the genus *Brucella*. *Brucella melitensis* and *B*. *abortus* cause disease mostly in small ruminants and cattle, respectively [18]. Although it displays a preferential host-range, *B*. *melitensis* can infect cattle in mixed breeding areas where it coexists with small ruminants [19]. Also, in West Africa *B*. *abortus* infection in small ruminants is noted to occur in areas where the animals are in contact with cattle and where *B*. *melitensis* is absent [20] [19]. Brucellosis is spread through contact with abortion products and vaginal fluids and by milk feeding, through semen or congenitally. Sheep can also be infected by *B*. *ovis*, a non-zoonotic species. *Brucella melitensis* and *B*. *abortus*, together with *B*. *suis* and *B*. *canis*, cause human brucellosis and contact with infected animals and consumption of raw dairy products is the most common source of transmission [21]. Brucellosis causes economic losses in livestock due to abortions and infertility.

National epidemiological surveys for brucellosis in Morocco in 1996 and 2010 showed that bovine brucellosis is more prevalent in the north-west coastal and central zones where the cattle density is the highest, with a mean individual and herd prevalence of 2.1% (n = 8991) and 4.9% (n = 1168), respectively (Government survey, 2010). Herd seroprevalences have remained at a similar level to those reported in 1977 (4.6%) and in 1988 (4.9%) (19). Initiatives to control brucellosis in Morocco have had varied success. A national vaccination campaign using S19 from 1989 to 1994 showed little impact on herd prevalence [22]. By contrast, a public-private initiative (2007) that included RB51 and/or S19 vaccination and test and slaughter on farms that are members of professional associations or cooperatives reduced brucellosis herd seroprevalence from 40% to 0.4% in member farms [23]. In total 81230 cattle were serologically tested for brucellosis, 55869 were vaccinated and 2901 were culled at a cost of $US 2.6 million. A large bacteriological study comprising 500 samples from 357 cattle isolated *B*. *abortus* biovar 1 and 3 in the 1980s [24,25].

Brucellosis in small ruminants is a recognized problem across the northern Mediterranean zone of Morocco and in inland mountainous areas where sheep and goat populations dominate. In the mid-1990s, small ruminant brucellosis emerged in in the Oriental region where Morocco shares a border with Algeria and prompted a programme of mass vaccination of small ruminants with the live attenuated vaccine *B*. *melitensis* Rev1 via the conjunctival route in the zone between 1997 and 2003. Premature ending of the vaccination campaign resulted in disease re-emergence and between 2009 and 2013 48 individual cases of small ruminant brucellosis were reported in the Northeastern region. A National survey in 2006 testing 11609 small ruminants yielded eight sero-positives, five of which were from the Oriental region (19). Bacteriological evidence of brucellosis in small ruminants in Morocco is limited to only three studies and they reported isolation of *B*. *melitensis* biovar 3 [26–28].

This study aimed to estimate the current prevalence of bovine tuberculosis in cattle, and the seroprevalence of the zoonotic *Brucella* species in cattle, sheep and goats, and to assess BTB and brucellosis risk factors in Sidi Kacem province in Morocco.

## Material and methods

### Study area

Sidi Kacem province comprises 29 communes and is sub-divided into two agro-hydrologic zones: “rainfed” (bour) in the northeast and “irrigated” in the southwest. The irrigated zone is characterized by low-lying plains, contrasting with the rainfed zone, which is mountainous (altitude from 150 to 500m). The number of cattle in the rainfed zone is 40% of the total cattle population of the province. The irrigated zone is dominated by an intensive mode of livestock rearing characterized by larger herd sizes (15 cattle/herd) [29] and European dairy breeds (Holstein-Friesian, Montbeliarde, Tarentaise) or cross-bred cattle. The livestock production system in the rainfed zone is more extensive, with dominance of local cattle breeds and smaller herd sizes (5 cattle/herd).

### Sampling

Sample size was determined using standard formulae for cluster surveys [30], we considered the village as a cluster, assuming the following parameters, i) a BTB prevalence of 18% [national survey 2004 [15]], ii) a mean number of cattle per household of 9, iii) an average number of 2 households selected per cluster and iv) an intra-class correlation coefficient of 0.2 [31,32]. An average of 62 villages (douars) and 124 households are required to estimate the prevalence with a precision (defined as one half-length of the 95% CI) of 5%-points. Clusters were randomly selected based on the official village lists available for Sidi Kacem province. Two households per village were randomly selected based on livestock-owning households provided by the chief of the commune upon arrival in each village; all cattle in cattle owning households were sampled. As small ruminant flock numbers were high, not all sheep and goats were sampled in small-ruminant owning households, a maximum of 20 animals were sampled per household.

### Herd and animal level data

**Fig 1:**
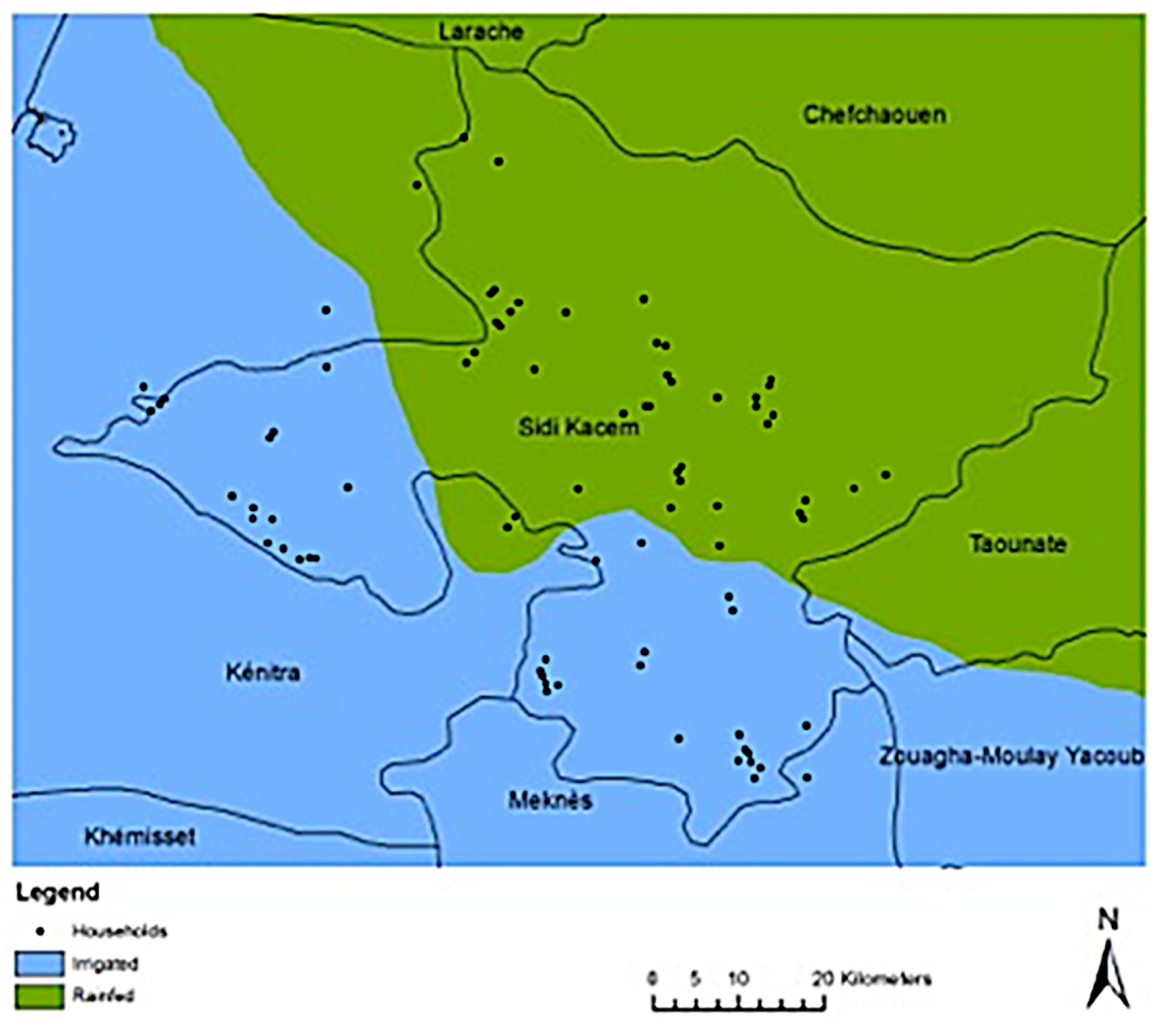
Sidi Kacem rainfed and irrigated regions and geo-localisation of the household screened.

At herd level, GPS coordinates (Fig 1.), livestock production system, grazing system and herd size were recorded.

Most sampled households owned cattle, but not small ruminants. Table 1 shows the number of households and the species present within the household. For every animal, age, gender, breed and body condition score (BCS) were recorded.

**Table 1:** Number of sampled households in terms of present species.

*Species owned by household*	*Number of households*
Cattle only	44
Sheep only	2
Goats only	0
Cattle sheep and goats	4
Cattle and sheep	75
Cattle and goats	0
Sheep and goats	0
**TOTAL**	125

### Diagnosis of bovine tuberculosis

The single intradermal comparative cervical skin test (SICCT) was used following OIE Terrestrial Manual standards for BTB screening [33]. Briefly, injection sites were clipped and cleaned; a fold of skin within each clipped area was measured with callipers and 0.2 ml each of bovine and avian purified protein derivative (PPD) (25000 IU/ml) was injected in the left neck region. Injections were performed using a separate intradermal gun for each PPD in two spots separated by approximately 15 cm. The skin-fold thickness at each injection site was re-measured 72h after injection by the same individual. Any exudate, oedema and pain observed in the injection sites were recorded. The SICTT was interpreted using the OIE recommended cut off and an animal was considered positive if the increase in skin thickness of the bovine PPD injection site was greater than the skin thickness of the Avian PPD injection site by 4 mm or more. The reaction was considered inconclusive if the difference was between 1 and 4 mm. The reaction was interpreted as negative if the increase in the skin thickness was equal for the bovine and avian PPD injection sites [33].

### Brucellosis sero-diagnosis and bacteriology

Blood from 1194 cattle and 1082 small ruminants across 125 households was collected (Fig 1) and all sera were stored in cool boxes before laboratory processing. After 24h storage at 4°C to allow serum separation, the tubes were centrifuged, and sera extracted, aliquoted and tested for antibodies to smooth *Brucella* using the Rose Bengal Test (RBT). There are two OIE accepted [34] variants of the test: the standard (sRBT) and the modified (mRBT) RBT that differ in the proportion of serum:antigen used: equal volumes (25–30 μl) in the sRBT and a higher volume of serum (75 μl serum and 25 μl antigen) in the mRBT. The mRBT increases the sensitivity when testing sera from small ruminants [35,36] but this modification is not recommended when testing cattle sera because it reduces the specificity in areas where S19 vaccination is implemented. Accordingly, the following testing strategy was used. Immediately after collection, one aliquot was used for screening of both cattle and small ruminant sera using the mRBT. This initial screening aimed to collect milk samples from seropositive lactating females for bacteriological investigations; using the mRBT increased the diagnostic sensitivity thereby maximising the number of milk samples collected. A second aliquot was stored at -18°C and sent to the “Instituto de Salud Tropical y Depto. Microbiología y Parasitología, Universidad de Navarra (UNAV)” for screening. Cattle samples were screened using the sRBT protocol and small ruminant samples were screened using sRBT and mRBT in parallel. The antigen used was a suspension of fully smooth *B*. *abortus* 1119 standardized according to international guidelines [34] and controlled for quality using a panel of brucellosis positive and negative serum samples [37].

Bacteriology was undertaken on milk samples obtained from 21 seropositive cattle (three CITA and three Farrell’s selective media plates for each sample) [38]. After incubation for 4–7 days at 37°C (5–10% CO_2_ atmosphere), suspicious colonies were re-plated on the same media for preliminary identification. Isolates were considered presumptive for *Brucella* by agglutination with anti-*Brucella* serum coated staphylococci and oxidase and urease tests [39]. Colonies found to be suspicious were stored at -20°C in vials of Triptic Soy Broth (TSB) with 5% DMSO for further typing at the WHO and OIE collaborating centres for the diagnosis of animal and human brucellosis at the “Universidad de Navarra (UNAV)” and “Centro de Investigación y Tecnología Agroalimentaria de Aragón (CITA)” in Spain. Isolates were confirmed as *Brucella* species and typed at species level using Bruceladder^®^ multiplex PCR [40] and, at biovar level, using classical phenotyping methods [39], i.e. oxidase and urease tests, CO_2_ requirement, agglutination with monospecific anti-A/anti-M sera, lysis with phages (Tb, Wb, Iz1 and R/C), sensitivity to dyes (fucsine, thionin and saphranine) and Crystal Violet exclusion test (to assess absence of dissociation) [39].

### Statistical analyses

Data were entered in Access 2012 and analysed using Stata 12.1. Generalized linear mixed models (GLMM) for binary outcomes were used to estimate prevalence and to identify risk factors. The GLMMs included a logit link function to estimate the odds ratios (OR) to quantify the relationship between different risk factors and the diseases [30]. Initial inspection of the variance components using multilevel models showed that most of the variance in the response is at household level rather than at village level and therefore we included households as random effect to account for clustering. Prevalence and corresponding confidence intervals were estimated fitting a constant only model. The predictors variables evaluated were livestock production system (categorized as intensive, semi-intensive, extensive), rainfed or irrigated grazing, age (up to 1 year, up to 3 years and older), sex, breed and body condition score (BSC, 1 to 2, 2.5 to 3, 3.5 +). Categories with very few observations, i.e. extensive production system and BSC above 3.5, were collapsed with their adjacent categories for the analysis. The multivariable model was pre-specified and included all predictor variables. Because the individual level brucellosis prevalence was low we had to exclude predictor variables without positive animals in one of their categories to avoid (quasi) complete separation. All inconclusive diagnostic test results were omitted prior analysis.

Considering that SICTT has a high specificity and an average sensitivity, we calculated both apparent and true individual prevalence. To calculate true prevalence, we used the Rogan Gladen-estimator [41] assuming a diagnostic sensitivity of 51.1% and a specificity of 98.9% as previously reported by Müller et al. from Chadian cattle using SICTT (42). The true individual of BTB prevalence was estimated as: {True prevalence = (Apparent prevalence+sp-1)/(sp+se-1)}. For brucellosis, given the high sensitivity and specificity of sRBT we can assume that apparent prevalence is close to true prevalence [42]. Sensitivity analysis used villages as clusters.

## Results

### Cattle characteristics

In total, 1201 bovines from 125 farms in 62 villages were examined of which 78.4% (n = 938) were female and 85% were crossbreeds (n = 1016), and almost all (96%, n = 1150) had a BCS equal to or less than 3. Most cattle (78%) (n = 936) were over 12 months. Cattle were almost equally distributed between the rain fed and the irrigated area, most from semi intensive herds (82.8%, n = 994) and only a few (n = 20) from extensive farming systems. Most herd sizes were below 15 (Table 2).

**Table 2:** Basic characteristics of 1201 cattle sampled in Sidi Kacem, Morocco.

*Characteristics*	*Classes*	*N (%)*
**Herd level**		
Livestock production system	Intensive	15 (12.2)
	Semi-intensive	105 (85.4)
	Extensive	3 (2.4)
Grazing system	Rainfed	58 (46.4)
	Irrigated	67 (53.6)
Herd size	1–15	92 (74.8)
	>15	31 (25.2)
**Individual level**		
Livestock production system	Intensive	187 (15.6)
	Semi-intensive	994 (82.8)
	Extensive	20 (1.6)
Grazing system	Rainfed	596 (49.6)
	Irrigated	605 (50.4)
Age (months)	0–12	265 (22.1)
	13–36	397 (33.1)
	>36	539 (44.8)
Sex	Female	938 (78.4)
	Male	258 (21.6)
Breed	Crossed	1016 (84.9)
	Imported	100 (8.4)
	Local	81 (6.7)
Body condition score	1–2	366 (30.5)
	2.5–3	784 (65.3)
	3.5–4	15 (1.3)

### Prevalence for bovine tuberculosis and brucellosis

Tuberculin skin test results were obtained for 1194 cattle. Of these, 107 animals were found inconclusive for BTB using the OIE interpretation criteria of the SICCT and were not included in the analysis. An individual apparent BTB prevalence of 20.4% (95% CI 18%-23%) and a herd prevalence of 57.7% (95% CI 48%-67%) were found for the remaining animals. Assuming a sensitivity of 51% and specificity of 99% as reported by Müller et al [43], a true individual BTB prevalence of 38.2% was estimated.

Brucellosis results using sRBT were available for 1179 cattle. Prevalence of bovine brucellosis was 1.9% (95% CI 1%-3%) at individual level and 9% (95% CI 5%-15%) at herd level. None of the 1044 sheep or 51 goats screened were sRBT positive and only 2/1044 sheep were mRBT positive.

### Risk factors analysis

#### a. Bovine tuberculosis

The uni- and multivariable analysis of BTB risk factors are shown in Table 3. Age higher than 36 months was significantly associated with a higher risk of BTB compared with age below 12 months (28.2% vs 13.7% OR: 2.6, 95% CI 1.4–4.8). Imported breeds were observed to have a lower risk of BTB compared to crossbreds (16.7% vs 21.7%, OR: 0.4,95% CI 0.1–0.8). We observed the lowest prevalence in local breeds (9.6%) but the confidence interval was broad and included unity (OR: 0.5 95% CI 0.2–1.4). Male animals showed a lower prevalence (12.0%) compared to female animals (22.7%) but this relationship was not statistical significant in the multivariable model (OR: 0.8 95% CI 0.4–1.4), likely due to the fact that only very few males were older than 36 months.

**Table 3:** Individual and herd risk factors of BTB in 1087 cattle.

*Risk factor*	*Classes*	*N screened*	*% (N positive)*	*OR (95% CI)*	*mOR (95% CI)*
**Herd level**					
Livestock production system	Intensive	170	34.7 (59)	Ref	—
	Semi-intensive & extensive	922	17.8 (164)	0.3 (0.1–0.9)	0.5 (0.2–1.4)
Grazing system	Rainfed	550	11.0 (60)	Ref	—
	Irrigated	544	30.1 (164)	3.5 (1.8–6.5)	3.1 (1.6–6.2)
Herd size (animals)	1–15	600	15.7 (94)	Ref	—
	>15	492	26.2 (129)	2.0 (0.9–4.1)	2.1 (1.0–4.3)
**Individual level**					
Breed	Crossed	918	21.7 (199)	Ref	—
	Imported	96	16.7 (16)	0.3 (0.1–0.7)	0.4 (0.1–0.8)
	Local	73	9.6 (7)	0.5 (0.2–1.3)	0.5 (0.2–1.4)
Age (months)	0–12	241	13.7 (33)	Ref	—
	13–36	367	14.4 (53)	1.4 (0.8–2.5)	1.2 (0.7–2.3)
	>36	475	28.2 (134)	3.9 (2.3–6.6)	2.6 (1.4–4.8)
Sex	Female	845	22.7 (192)	Ref	—
	Male	241	12.0 (29)	0.3 (0.2–0.6)	0.8 (0.4–1.4)
Body condition score	1–2	325	29.5 (96)	Ref	—
	2.5–4	732	16.7 (122)	0.6 (0.4–0.8)	0.8 (0.5–1.3)

mOR: multivariable analysis OR

Ref: Reference category

At herd level, irrigated grazing systems showed a significantly higher risk of BTB compared with rainfed systems (30.1% vs 11%, OR: 3.1 95% CI 1.6–6.2). Large herd size showed a higher risk of BTB than small to medium herd size (26.2% vs 15.7%, OR: 2.1, 95% CI 1.0–4.3).

#### b. Brucellosis

Uni- and multivariable analysis of risk factors for brucellosis are shown in Table 4. Due to the low number of positive brucellosis cases, the study was not sufficiently powered to detect statistically significant differences.

**Table 4:** Individual and herd risk factors of bovine brucellosis in 1177 cattle.

*Risk factor*	*Classes*	*N screened*	*% (N positive)*	*OR (95% CI)*	*mOR (95% CI)*
**Herd level**					
Livestock production system	Intensive	187	6.9 (13)	Ref	--
	Semi-intensive & extensive	994	0.9 (9)	0.3 (0.05–2.02)	0.6 (0.1–4.3)
Grazing system	Rainfed	595	0.5 (3)	Ref	--
	Irrigated	586	3.2 (19)	4.7 (0.8–26.7)	3.4 (0.6–21.0)
Herd size (animals)	1–15	643	0.77 (5)	Ref	--
	>15	538	3.16 (17)	2.8 (0.5–15.1)	2.3 (0.5–11.9)
**Individual level**					
Breed	Crossed	997	2.2 (22)	Ref	--
	Imported	100	0.0 (0)	Nd	--
	Local	80	0.0 (0)	Nd	--
Age (months)	0–12	257	0.4 (1)	Ref	--
	13–36	388	0.3 (1)	0.5 (0.03–8.5)	0.4 (0.02–7.41)
	>36	527	3.8 (20)	6.9 (0.8–60.4)	5.1 (0.6–45.6)
Sex	Female	928	2.4 (22)	Ref	--
	Male	248	0.0 (0)	Nd	--
Body condition score	1–2	363	4.7 (17)	Ref	--
	2.5–4	783	0.6 (5)	0.2 (0.05–0.7)	0.5 (0.1–1.8)

mOR: multivariable analysis OR

Ref: Reference category

Nd: Not determined because of perfect prediction

We observed a lower prevalence in animals with a BCS lower than 2 (0.7% vs 4.7%), and a higher prevalence in irrigated grazing systems (3.2% vs 0.5%) and in large herds (3.2% vs 0.8%).

### Characterization of *Brucella* isolates

Three *Brucella* strains isolated from female cattle were identified as *B*. *abortus* biovar 1 through classical typing. Isolates were from cows belonging to the same herd located in the irrigated zone. The cows were over 72 months old; two had a history of abortions and BCS of less than 2.

## Discussion

The overall BTB individual apparent prevalence in this study was 20.4%, similar to that reported in 2004 (18%) during national tuberculin skin testing. However, given the reported low sensitivity of BTB testing these figures are likely to be underestimated. The BTB 57.7% herd prevalence in this study is higher than that previously reported at 33% in 2004 at national level [15]. Despite that the 2004 survey applied the single intradermal tuberculin skin test (SITT), which is less specific than the SICTT used in the present study. While the single tuberculin skin test (SITT) may result in a high number of false positives, the comparative tuberculin skin test (SICTT) was shown to reduce false positives and cross reactions (i.e. the single test has a higher diagnostic sensitivity and the comparative test is more specific) [44]. The BTB prevalence in the present study is also far higher than those reported in Uganda (6%) [45], Niger (3.6%) [46], rural Ethiopia (5.5%) [47], and Tanzania (2.4%) [48]. The high prevalence in Morocco may be explained by more intensive dairy cattle farming practices (see below), similar to Central Ethiopia, where a herd prevalence of 50% was registered in 2012 using SICTT [49]. On the other hand, BTB prevalence in Morocco (20.4%) was lower than that reported in Zambia (49.8%) and Mozambique (39.6%) [50,51].

The gold standard for brucellosis diagnosis is isolation and identification of *Brucella* spp. However, bacteriological culture is cumbersome, expensive and requires skill and facilities rarely available in resource-poor countries and consequently, indirect testing of anti-*Brucella* antibodies in serum is commonly applied for brucellosis screening. In this work, brucellosis apparent seroprevalence was examined using the sRBT for cattle and the mRBT for small ruminants. A rigorous meta-analysis of diagnostic tests for bovine brucellosis [52] based on solid data gathered by a panel of experts [53] has shown that the sRBT performs with diagnostic sensitivity and specificity values of 98.1% (95% CI [96.8%-99.1%]) and 99.8% (95% CI [99.7%-99.8%]), respectively, where vaccination is not practiced. Accordingly, and taking into account that vaccination was not undertaken in the area, no further serological testing by the so-called complementary tests was necessary. This is worth commenting upon because there are a number of works assuming that RBT lacks specificity in the absence of vaccination and needs to be confirmed by a second test. The above-cited meta-analysis and a recent rigorous analysis of publications meeting strict scientific criteria [42] prove that this is a misconception, possibly resulting from a misinterpretation of the use of tests in programs where eradication through vaccination in parallel to test and slaughter is applied (for a discussion see [42,54]). This is also true for small ruminants testing where optimal sensitivity is obtained using the mRBT [35]. Indeed, mRBT has been shown to be useful for reducing the cost and the time of brucellosis screening in *B*. *melitensis* eradication campaigns [55]. The standard serum agglutination test is not recommended by OIE due to its low sensitivity, and the complement fixation test is technically cumbersome and does not outperform RBT in terms of specificity and sensitivity in the absence of vaccination [35,42]. Indirect ELISA shows similar sensitivity and specificity to RBT in the absence of vaccination, but is more expensive [52] and need to be validated in the target population since its diagnostic performance depends on the manufacturer and the selection of an adequate cut-off to discriminate positive and negative samples [56] [42]. Despite original reports [57], recent studies show that competitive ELISAs do not have optimal sensitivity [58]. Thus, as Morocco is a country where no official brucellosis vaccination is currently practiced, sRBT and mRBT can be recommended for serological surveys in cattle and small ruminants respectively.

Using these methods, individual apparent brucellosis prevalence found in this work in cattle (1.9%) is near the 2.1% reported for the national survey of 2010–2011. National statistics from 1982 to 1992 registered a mean herd prevalence ranging from 2.1% to 4.9%, which is lower than brucellosis herd prevalence (9%) found in the present study [26]. A recent study using mRBT to investigate bovine brucellosis in 25 farms (221 cattle) reported a prevalence of 33.48%, which is higher than that reported by our study [59]. However, the high prevalence could have been due to the irrigated study area and the targeted animals (females older than 18 months). Individual brucellosis prevalence found in Sidi Kacem was also lower than that found in Egypt (23.8%) [60], Uganda (5%) [61], Nigeria in 2011 (4.04%) [62] and Ethiopia in 2006 (2.9%) [63]. The *Brucella* strains isolated from the three Friesian/Holstein cows and belonging to the same herd in the irrigated zone were found to be *B*. *abortus* biovar 1, and phylogenomic studies (not described here) show that these strains have some homogeneity with Spanish strains. This is in line with a study conducted in the mid-1980s yielding 8 *B*. *abortus* biovar 1 and 28 *B*. *abortus* biovar 3 strains from 500 samples [24,25]. A subsample of these Moroccan strains was examined as part of a study characterising *B*. *abortus* strains of African origin and found the 12 Moroccan strains to be identical to *B*. *abortus* biotypes isolated in Europe.

In this study only two sheep from two different herds in the irrigated area showed a positive mRBT result. The mRBT positive sheep were from a herd that had several cases of cattle brucellosis. Livestock from this village grazed on shared pasture and the sheep may have developed seropositivity due to contact with *B*. *abortus* infected cattle. These sheep could have developed antibodies but cleared the infection and hence be of negative infection status or alternatively be infected and a source of contagion to other ruminants or humans. A study performed in Northern Morocco and the Middle Atlas in 2013, investigated brucellosis among 23 sheep and goat herds using mRBT. The results of the study showed an individual and herd prevalence of 13.3% and 43% respectively. The high prevalence registered could be explained by the condition of animals sampled, which were exclusively aborting females, so abortion could be considered as a confounding factor for that study [64]. Very little is known about the course of infection by *B*. *abortus* in sheep, which seems to be a very rare event even in areas free of *B*. *melitensis* and where cattle are infected by *B*. *abortus* [65]. Regrettably, slaughter for collection of necropsy samples for bacteriological confirmation were beyond the scope of this study. Further investigations are necessary to clarify the epidemiology of small ruminant brucellosis in the areas targeted by the study.

Consistent with a recent review [9], we observed a higher BTB and brucellosis prevalence in intensive livestock production systems, although the relationships were not statistically significant. Intensive dairy farming conditions within a confined environment with less access to sun, air flow and high humidity conditions may enhance transmission of *Brucella* and *Mycobacteria* between animals. The higher stocking density, larger herd sizes, and propensity to buy in animals, together with higher calving frequency in intensive systems may also increase the risk of disease transmission. On the other hand, extensive systems, where animals are grazed in lower density on communal pasture, may have a lower risk of transmission because of the effect on the sun and heat on *Brucella and Mycobacteria* environmental contaminants [19]. The shift to a more intensive livestock production observed in rapidly urbanizing developing countries such as Morocco, could lead to an increase of zoonotic diseases transmission in the absence of animal recording systems, movement controls and well managed veterinary services [19,66].

As has been previously reported, Indigenous animals showed a lower risk of BTB, [51] compared to imported and cross breed animals. This may be explained by their non-adaptation to local conditions. Local indigenous breeds may not be a protective factor, however, because in Morocco extensive herds are dominated by local and cross-bred cattle, while intensive herds are dominated by imported breeds. Consequently, the breed could be a confounding factor for the production system [18]. Older animals showed a higher risk of BTB and bovine brucellosis as described in other studies [67]. All positive brucellosis cases were found to be females, but it must be interpreted in light of the fact that the sample was composed predominantly of female cattle. In Nigeria, females were described to be more susceptible to *B*. *abortus* infection [68]. In the present study, a low BCS showed a higher risk of BTB. BCS has previously been linked with BTB infection [69,70], although recent studies in Ireland and Tanzania showed no evidence of the association of low BCS and high BTB prevalence [71,72]. Low BCS could be linked to clinically advanced BTB [73], but in this study the initial status of the sampled animals was unknown.

Based on the findings of this study we conclude that bovine tuberculosis and brucellosis are prevalent in Sidi Kacem. Considering the economic losses caused by BTB and brucellosis, in addition to their public health impact, additional efforts should be deployed to design an integrated control strategy. Test and slaughter has been shown to be the most efficient elimination strategy for BTB in several countries. Many factors contribute to the success of a control and elimination campaign. Trust between all stakeholders especially the farming industry, the government and the farmers are a very important component which contributed to the success of brucellosis and BTB control program in other contexts. Correct application of livestock biosecurity measures, early diagnosis of the disease, and application of movement restrictions also affect the success of a control campaign [74]. However, in Morocco, relationships between livestock keepers, local authorities and veterinary services are characterized by mistrust. Solid and sustainable control cannot be achieved without the conviction and participation of all stakeholders. Sensitisation and education campaigns of all stakeholders are required to improve adherence to and acceptance of control programs by local populations and decision makers. A rigorous application of the decided strategy and involvement of the animal owners in the decision process are pivotal for the success of control and elimination programs [75].

The best strategy for controlling brucellosis in Morocco would be conjunctival mass vaccination every two years just before the natural breeding season or immediately after calving/lambing/kidding. This is the best option as prevalence is high and the veterinary services are not able to apply individual tagging allowing vaccination of young replacements only [76]. However, vaccination needs to be sustained over time to be effective. Premature removal of S19 or Rev1, or replacement with a less effective vaccine e.g. RB51, has led to failure of previous attempts for control in Morocco (19).

A BTB transmission model for Morocco indicated that BTB could be controlled within 25 years, if 50% of cattle were tested annually, and infected animals were slaughtered at an estimated cost of 1.55 billion Euros [77]. Taking into consideration the current infectious diseases prioritized by Morocco (e.g: foot and mouth disease, sheep pox virus and Peste des Petits Ruminants [PPR]), such a control strategy is currently deemed unaffordable for a middle-income country. Wildlife reservoirs can also complicate control operations. The presence of a wildlife reservoir (e.g. badgers in Ireland) has caused reemergence of BTB in developed countries [78]. Wildlife reservoirs have been shown to exist in Africa (feral baboons in Kenya [79] and warthog and buffalo in Uganda [80]). The role of a wildlife reservoir in BTB transmission in Morocco is unknown, and other barriers to control are considered more critical including: lack of efficient organization of veterinary services; prioritization of other highly infectious viral and parasitic diseases; limited technical capacity and financial constraints [81].

As for other neglected zoonosis, evidence and advocacy is necessary to convince policy-makers and communities of the benefits of disease control [82,83]. The evaluation of the cost of BTB should take into account the cost of the human disease, and for this purpose an investigation of the prevalence of *M*. *bovis* in humans is required, as well as a calculation of the direct and indirect cost of a human TB case. Cross-sectoral socio-economic analysis of the cost of both diseases is needed. In addition, support from private industry (e.g. the milk industry) could sustain BTB and brucellosis control campaigns, as they will also benefit from the control measures. Novel methods for innovative financing should be examined to mobilize investment for interventions that contribute towards elimination of neglected zoonosis [84,85] and benefit human and animal health.

The present study confirms that there is added value in investigating multiple zoonoses simultaneously, especially for zoonoses with a reservoir overlap. Undertaking brucellosis and BTB screening in parallel and in multiple hosts is logistically and technically feasible. The added value of an integrated approach to epidemiological investigations on zoonoses has been demonstrated in Chad [86]. Morocco could consider a parallel elimination campaign for BTB and brucellosis that optimizes use of human and economic resources.

## Ethical clearance

The methodology of the study including the household questionnaire was reviewed and validated by international and national research expert partners within the ICONZ project. In addition, the Moroccan veterinary services (ONSSA) approved and granted authorization for the study. The purpose of the study was explained to the local authorities and to the farmers. The tests used for the screening of BTB and brucellosis are routinely used by the veterinary services of Morocco.

## Supporting information

S1 Dataset HouseholdSurvey_ICONZ_2012. (XLSX)

S2 Dataset ICONZ_Sidi Kacem_Database_Prevalence. (XLSX)

S3 Dataset Serology_Brucellosis_MoroccoICONZ. (XLSX)
